# Inhibition of Survivin Homodimerization Decreases Neuroblastoma Cell Growth

**DOI:** 10.3390/cancers15245775

**Published:** 2023-12-09

**Authors:** Carmen Dorneburg, Celimene Galiger, Giovanna L. Stadler, Mike-Andrew Westhoff, Volker Rasche, Thomas F. E. Barth, Klaus-Michael Debatin, Christian Beltinger

**Affiliations:** 1 Department of Pediatrics and Adolescent Medicine, University Medical Center Ulm, 89075 Ulm, Germany; carmen.dorneburg@uni-ulm.de (C.D.); giovanna.stadler@uniklinik-ulm.de (G.L.S.); mike-andrew.westhoff@uniklinik-ulm.de (M.-A.W.); klaus-michael.debatin@uniklinik-ulm.de (K.-M.D.); 2 Department of Internal Medicine II, University Medical Center Ulm, 89075 Ulm, Germany; volker.rasche@uni-ulm.de; 3 Department of Pathology, University Medical Center Ulm, 89075 Ulm, Germany; thomas.barth@uniklinik-ulm.de

**Keywords:** survivin, BIRC5, neuroblastoma

## Abstract

**Simple Summary:**

Elevated expression of BIRC5/survivin, a protein crucial for cell division, is linked to unfavorable outcomes in neuroblastoma (NB), a prevalent pediatric tumor. Novel therapeutic approaches are consequently warranted. Therefore, we examined the effects of S12 and LQZ-7I, small molecules that disrupt survivin’s interaction with itself and other proteins, on NB cells in vitro and in vivo. We demonstrate that these drugs effectively suppress the growth of NB cells, partly by hindering cell division. In contrast, incomplete inhibition of BIRC5 transcription did not diminish NB growth. We conclude that inhibitors of survivin protein interaction hold promise as a novel therapeutic class against NB and should be further investigated.

**Abstract:**

Increased expression of BIRC5/survivin, a crucial regulator of the mitotic spindle checkpoint, is associated with poor prognosis in neuroblastoma (NB), the most common extracranial tumor of childhood. Transcriptional inhibitors of survivin have been tested in adult cancers and inhibitors of survivin homodimerization are emerging. We compared genetic inhibition of survivin transcription with the inhibition of survivin homodimerization by S12 and LQZ-7I, chosen from a larger panel of survivin dimerization inhibitors with activity against NB cells. Mice hemizygous for *Birc5* were crossed with NB-prone *TH-MYCN* mice to generate *Birc5*+/-/*MYCNtg*/+ mice. The marked decrease of survivin transcription in these mice did not suffice to attenuate the aggressiveness of NB, even when tumors were transplanted into wild-type mice to assure that immune cell function was not compromised by the lack of survivin. In contrast, viability, clonogenicity and anchorage-independent growth of NB cells were markedly decreased by S12. S12 administered systemically to mice with subcutaneous NB xenotransplants decreased intratumoral hemorrhage, albeit not tumor growth. LQZ-7I, which directly targets the survivin dimerization interface, was efficacious in controlling NB cell growth in vitro at markedly lower concentrations compared to S12. LQZ-7I abrogated viability, clonogenicity and anchorage-independent growth, associated with massively distorted mitotic spindle formation. In vivo, LQZ-7I effectively reduced tumor size and cell proliferation of NB cells in CAM assays without apparent toxicity to the developing chick embryo. Collectively, these findings show that inhibiting survivin homodimerization with LQZ-7I holds promise for the treatment of NB and merits further investigation.

## 1. Introduction

Neuroblastoma (NB), the most common extracranial solid tumor of childhood, still carries a poor prognosis in high-risk patients, despite remarkable progress in the understanding, stratification and therapy of this tumor [1,2,3].

Survivin (BIRC5), discovered 25 years ago [4], is a eukaryotic protein essential for mitosis and inhibits apoptosis, constitutes a cancer-associated effector of cellular metabolism [5] and is involved in the migration, angiogenesis and stemness of cancer cells [6]. Survivin is highly expressed in embryonic and fetal organs, as well as in the majority of cancers, but is undetectable in most adult non-proliferating tissues [6]. Survivin is overexpressed in NB [7], where 17q25, the chromosomal locus of BIRC5, is frequently gained [8]. Overexpression of survivin in NB is associated with poor-prognosis features [9,10] and knock-down of survivin induces apoptosis in NB cells [11].

Survivin consists of 142 amino acids with a BIR domain and a central linker region. The linker region assembles survivin into homodimers that are resilient against proteasomal degradation and mediate its antiapoptotic function [6]. The linker region also forms survivin heterodimers essential for mitosis [12,13,14,15,16] and other functions.

Given its pivotal roles in cancer, survivin is an attractive potential therapeutic target. Efforts have been undertaken to decrease its transcription, to degrade its mRNA or to disrupt its homo- and heterodimers [17]. Survivin vaccination constitutes another therapeutic approach [18,19] and has been investigated in a NB mouse model [20].

The most extensively studied anti-survivin drug is YM155. Initially described to specifically suppress survivin transcription [21], additional mechanisms have been proposed [22,23,24,25,26,27,28]. After promising phase I trials, the results of phase II studies in various cancer types disappointed [22,23,24,25]. Antisense oligonucleotide approaches to degrade survivin mRNA also failed [17].

Much effort is directed at developing inhibitors of survivin protein interactions. Among these is the small molecule S12, a lead compound identified by screening and designed to target a cavity adjacent to the survivin dimerization interface to inhibit homodimerization of survivin [29]. S12 alters spindle formation, induces mitotic arrest and causes cell death of cancer cell lines [29,30]. In vivo, S12 inhibits tumor growth of pancreatic cancer cells [29].

More recently, LQZ-7, a small molecule directly targeting the survivin dimerization interface, has been found using in silico screening [31]. LQZ-7 inhibits homodimerization of survivin, thus causing its proteasomal degradation [31]. LQZ-7 was modified to generate LQZ-7I, which exhibited improved efficacy [32]. LQZ-7I induced proteasome-dependent degradation of survivin, caused apoptosis in various cancer cell lines at low micromolar concentrations and inhibited the growth of prostate cancer cells in mice [32].

Given the critical role of survivin in maintaining neuroblastoma (NB) cells, and the lack of survivin-targeting drugs that have progressed to clinical trials for NB, we investigated the efficacy of survivin-targeting strategies in NB. To evaluate the impact of incomplete survivin inhibition, we developed a transgenic mouse model of Birc5 heterozygosity in NB. In subsequent pilot in vitro experiments, we explored the anti-NB efficacy of the survivin homodimerization inhibitors S12, LQZ-7F, and LQZ-7I in comparison to the survivin transcription inhibitor YM155 and the survivin heterodimerization inhibitor LLP-3 [17,33,34,35]. Focusing on homodimerization inhibitors, we selected S12 and LQZ-7I for further in-depth analysis based on their efficacy in the pilot experiments. Our findings show that inhibiting survivin homodimerization with LQZ-7I is a promising strategy for controlling NB cell growth.

## 2. Materials and Methods

### 2.1. Mice

Swiss:129sv survivin+/- [36] mice were obtained from the Mario Negri Institute for Pharmacological Research, Milano, Italy and were backcrossed with 129X1/SvJ mice for at least seven generations resulting in the mouse strain 129X1/SvJ;swiss *Birc5*+/-. 129X1/SvJ-Tg(*ratTH-hNMyc*)41WAV mice were obtained from the NIH, Bethesda, MD, USA. Six-week-old female immunodeficient athymic nude mice from Charles River were used for treatment experiments. The animal study protocol was approved by the Institutional Review Board of Regierungspräsidium Tübingen (protocol code TV-Nr. 1210 and date of approval 12.02.2015).

### 2.2. Small Animal Magnetic Resonance Imaging

Mice with palpable tumors (i.e., about 0.3 cm^3^) were subjected to MR analysis using a 11.7 Tesla small animal magnetic resonance scanner (BioSpec 117/16, Bruker, Ettlingen, Germany). Animals were analyzed 3 days per week until tumors reached a volume of 1.75 cm^3^.

### 2.3. Survivin Inhibitors

S12 inhibitor and YM155 were purchased from Calbiochem, Merck, Darmstadt, Germany. LLP3 was purchased from Sigma Aldrich and LQZ-7F from Glixx labs, Hopkinton, MA, USA. LQZ-7I was obtained from Biorybt, Cambridge, UK. All compounds were dissolved in DMSO.

### 2.4. S12 Therapy In Vivo

A total of 2 × 10^6^ Kelly NB cells resuspended in 100 µL of 100% matrigel (BD Biosciences, Heidelberg, Germany) were subcutaneously injected into 6-week-old athymic mice. One week after injection, mice bearing tumors were randomly divided into treatment and control groups. Mice were treated three times per week with intraperitoneal injections of 30 mg/kg S12 dissolved in PBS with 40% polyethylene glycol (PEG, Merck, Darmstadt, Germany), while the control group received PBS with 40% PEG. Mice were monitored regularly. Tumor volume was determined using a caliper [*v* = ½ (*W* × *W* × *H*)]. Doubling time was calculated as tumor volume change over time. Mice were sacrificed when tumors reached 1.5 cm in diameter.

### 2.5. Hematological Toxicity Analysis

Six-week-old female immunodeficient athymic nude mice were injected intraperitoneally with 30 mg/kg S12 three times per week for 4 weeks. Control animals received the vehicle only (PBS with 40% PEG). Blood samples were collected from the tail vein and hematological analysis was performed using a Hemavet 950 FS hematology analyzer (Drew Scientific, Miami, FL, USA).

### 2.6. Quantitative Real-Time PCR

Tumor tissue samples were collected from organs and frozen in liquid nitrogen. Tissues were cut into small pieces, covered with Trizol (Invitrogen, Thermo Fisher Scientific, Waltham, MA, USA) and placed in lysing matrix tubes with ceramic spheres (MP Biochemicals, Eschwege, Germany). Tissue Lyser (Qiagen, Hilden, Germany) with pulses for 20 s was used to disrupt and homogenize the solution. Subsequently, RNA was isolated using Direct-zol RNA Miniprep (Zymo Research, Freiburg, Germany). Total RNA was treated with DNaseI (Invitrogen). SuperScript III RT Kit (Invitrogen) was employed for subsequent cDNA preparation. qRT-PCR was performed with the CFX ConnectTM Real-Time system (BioRad, Feldkirchen, Germany) using the primers Birc5 fw caggggagtgctttctatgc and rev-gaggctggcttcatccact, and SsoAdvanced™ Universal SYBR^®^ Green Supermix (BioRad).

### 2.7. Western Blot and Antibodies

Tumor tissue samples were collected from organs and frozen in liquid nitrogen. Tissues were cut into small pieces, covered with RIPA lysis buffer and placed in lysing matrix tubes with ceramic spheres (MP Biochemicals). Tissue Lyser (Qiagen) with pulses for 20 s was used for homogenization. NB cells were harvested and lysed with RIPA lysis buffer. Protein concentrations were determined using a BCA Kit (Thermo Fisher Scientific). A total of 20 µg protein per sample was loaded. The blotting system iBlot (Thermo Fisher Scientific) was used according to standard protocols. After blocking with non-fat milk, membranes were incubated with the primary antibodies anti-mouse survivin (TIAP, Merck, Millipore, Burlington, MA, USA), anti-human survivin (R&D Systems, Minneapolis, MN, USA), anti-human actin (Sigma Aldrich, St. Louis, MO, USA) or anti-GAPDH (HyTest Ltd., Turku, Finland). Goat anti-mouse IgG-HRP (Santa Cruz, Heidelberg, Germany) was used as a secondary antibody.

### 2.8. Immunocytochemistry to Quantify Abnormal Mitotic Figures

NB cells were seeded onto cover slips in 24-well plates and treated with inhibitors. Formalin-fixed NB cells were probed with mouse anti-alpha-Tubulin (Calbiochem), secondary anti-mouse IgG Alexa Fluor 594 and Hoechst 33258 (Invitrogen). Fluorescence microscopy using a Keyence BZ-9000 microscope (Keyence, Neu-Isenburg, Germany) and Keyence Image analyzer software was performed. Mitotic figures were counted at 20× magnification in four visual fields per slide and the percentage of abnormal figures was calculated.

### 2.9. Chorioallantoic Membrane (CAM) Assay

A total of 1.5 × 10^6^ SK-N-AS cells were resuspended in a 1:1 mixture of serum-free DMEM medium and Matrigel^®^ (BD Biosciences, Franklin Lakes, NJ, USA) and transplanted onto the CAM of one-week-old, fertilized chicken eggs obtained from LSL Rhein-Main (Dieburg, Germany). One day after seeding, topical treatment was initiated with LQZ-7I at concentrations of 10 µM and 20 µM in 15 µL of PBS containing 0.1% DMSO. PBS and 0.1% DMSO were used as controls. Treatment was administered twice daily for three consecutive days. Four days after seeding, the tumors and surrounding CAMs were excised, formalin-fixed, paraffin-embedded and sectioned into 3 µm-thick slices. Sections were stained with haematoxylin (Merck KGaA, Darmstadt, Germany) and eosin (Sigma-Aldrich, St. Louis, MO, USA), and for Ki-67 (1:100, #M7240, Dako Deutschland GmbH, Hamburg, Germany). The immunohistochemical staining is detailed in the Supplementary Materials and Methods.

### 2.10. Statistical Analysis

Data were analyzed using GraphPad Prism 8.4.3 software (RRID:SCR_002798, GraphPad Software, San Diego, CA, USA) and are presented as means ± SD. For in vitro analyses no prespecified effect size was required. Three independent experiments were performed, unless stated otherwise. An appropriate sample size for animal experiments was determined by biometric calculation. The unpaired two-sided Student’s t test was used for comparison of two groups. One-way ANOVA was employed for multiple group comparisons. The log-rank (Mantel–Cox) test was used for survival studies. Results were considered significant if *p* < 0.05.

## 3. Results

### 3.1. The Hemizygous Deletion of Birc5 Does Not Decrease Aggressiveness of NB in TH-MYCN Transgenic Mice

**Figure 1:**
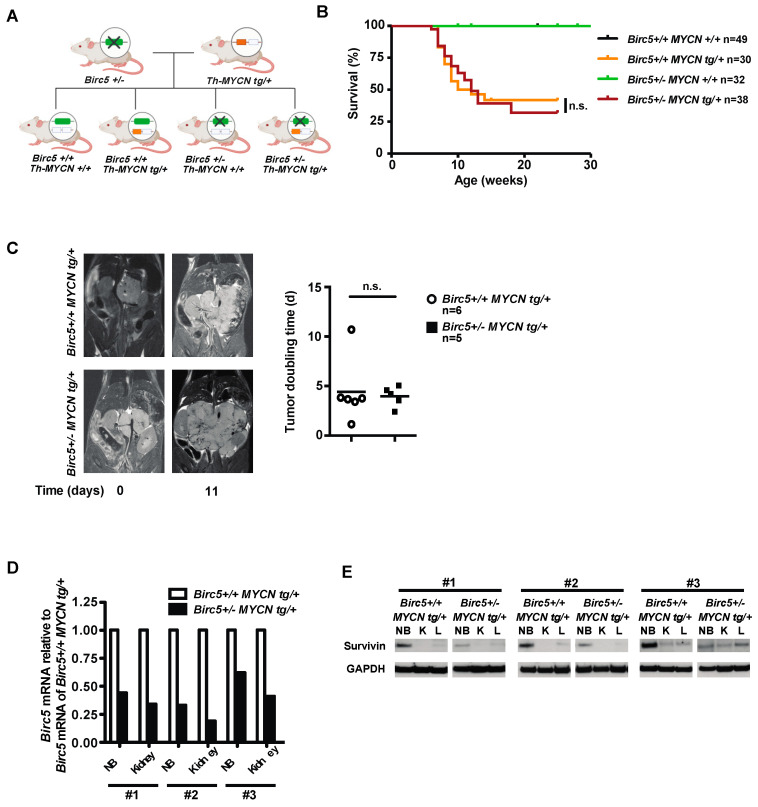
Survival of Birc5+/- MYCN tg/+ mice is not decreased compared to Birc5+/+ MYCN tg/+ mice. (**A**) Generation of Birc5+/- MYCN tg/+ mice. Heterozygous 129X1/SvJ;swiss Birc5 (Birc5+/-) and 129X1/SvJ-Tg(ratTH-hNMyc)41WAV (MYCN tg/+) mice were crossed. The four different genotypes of the offspring are depicted. (**B**) Birc5 hemizygosity does not decrease survival of tumor-bearing mice. Mice were monitored regularly for tumor development by palpation and were sacrificed when appearing moribund. Shown are the Kaplan–Meier survival curves of the respective genotypes. Statistical analysis was performed using the log rank test. n.s., not significant. (**C**) Tumor doubling time is not increased in Birc5 hemizygous tumors. Tumor growth in mice was monitored by MRI, shown are representative coronal MRI scans. Tumor volumes were determined for each time point and tumor-doubling times were calculated. Statistical analysis was performed using the Mann–Whitney test. n.s., not significant. (**D**) Hemizygous deletion of Birc5 effectively decreases Birc5 mRNA levels. RNA was isolated from tumors (NB) and kidneys of Birc5+/+MYCN tg/+ and Birc5+/- MYCN tg/+ mice. mRNA expression levels were determined by qRT-PCR. Shown are the results from three pairs of littermates (#1, #2 and #3). (**E**) Hemizygous deletion of Birc5 markedly decreases survivin protein levels. Proteins were isolated from tumors (NB), kidneys (K) and livers (L) of Birc5+/+MYCN tg/+ and Birc5+/- MYCN tg/+ mice. Survivin protein levels were determined using a Western blot. Shown are the results from three pairs of littermates (#1, #2 and #3). Original western blots are presented in File S1.

First, we modeled the effects of decreased survivin transcription in NB and normal tissues in the mouse to simulate the clinical effects of survivin transcription inhibitors against NB. Survivin is essential for the survival of normal proliferating cells, including hematopoietic and immune cells. We therefore reasoned that marked, yet incomplete inhibition of survivin transcription would best mirror what can safely be achieved in patients. Thus, we generated a novel mouse model, where *Birc5* is hemizygously deleted in all tissues [36] and MYCN is overexpressed in the peripheral sympathetic nervous system for induction of NB [37]. To this end, *Birc5+/*- mice were crossed with *TH-MYCNtg*/+ mice to generate *Birc5+/*-/*MYCNtg*/+ mice (Figure 1A). Surprisingly, *Birc5*+/-/*MYCNtg*/+ and *Birc5+*/+/*MYCNtg*/+ mice developed NB with similar incidence and latency (Figure 1B). In addition, there was no difference in tumor doubling times calculated from consecutive MRI scans (Figure 1C). Histologically, tumor proliferation, apoptosis and angiogenesis were not altered by the decreased survivin levels in the NB of *Birc5+/-/MYCNtg*/+ mice (Supplementary Figure S1).

The expression of survivin was determined in the NB and the non-tumorous kidneys of *Birc5+/*-/*MYCNtg*/+ and *Birc5+*/+/*MYCNtg*/+ mice. Hemizygosity in *Birc5* markedly decreased survivin mRNA in the kidneys and, importantly, also in the NB (Figure 1D). This resulted in a pronounced reduction of survivin protein levels in hemizygous NB (Figure 1E).

These results show that a marked, yet incomplete decrease of survivin transcription does not suffice to decrease the aggressiveness of NB in *Birc5+/*-/*MYCNtg*/+ mice.

### 3.2. Retained Aggressiveness of NB in Birc5+/- MYCN tg/+ Mice Is Not Caused by Decreased Survivin in Non-Malignant Tissues

**Figure 2:**
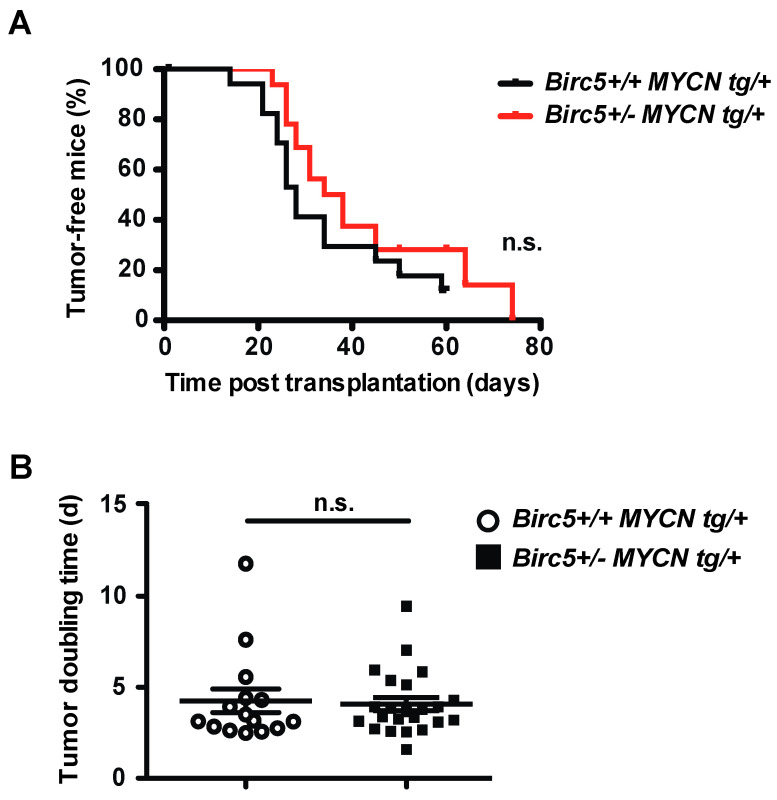
Aggressiveness of NB from Birc5+/- MYCN tg/+ mice is not decreased when transplanted into wild-type mice. (**A**) Unaltered tumor incidence and latency. Tumors from Birc5+/+ MYCN tg/+ (n = 4) and Birc5+/- MYCN tg/+ mice (n = 6) were obtained and 1 × 10^6^ tumor cells were transplanted subcutaneously into at least n = 4 syngeneic Birc5+/+ MYCN+/+ mice. Shown are Kaplan–Meier curves of tumor-free mice of the respective genotype. Statistical analysis was performed using the log rank test. n.s., not significant. (**B**) Unaltered tumor growth. Tumor size was measured regularly using a caliper until mice were sacrificed when tumors reached 1.5 cm in diameter. Shown are tumor doubling times from individual recipient mice. Statistical analysis was performed using the *t*-test. n.s., not significant.

The marked, yet incomplete decrease of survivin transcription in the *Birc*5+/- *MYCN tg*/+ mice did not attenuate the aggressiveness of NB. This could be explained by reduced expression of survivin in immune cells, decreasing their proliferation, function and survival [38,39,40]. We therefore transplanted NB that arose in *Birc*5+/- *MYCN tg*/+ mice into wild-type mice. However, the aggressiveness of NB from *Birc*5+/- *MYCN tg*/+ mice was not decreased when transplanted into wild-type mice (Figure 2A,B). Thus, the retained aggressiveness of NB in *Birc*5+/- *MYCN tg*/+ mice is not caused by decreased survivin in immune cells.

### 3.3. The Inhibitors of Survivin Protein Interactions Decrease Viability of NB Cells

**Figure 3:**
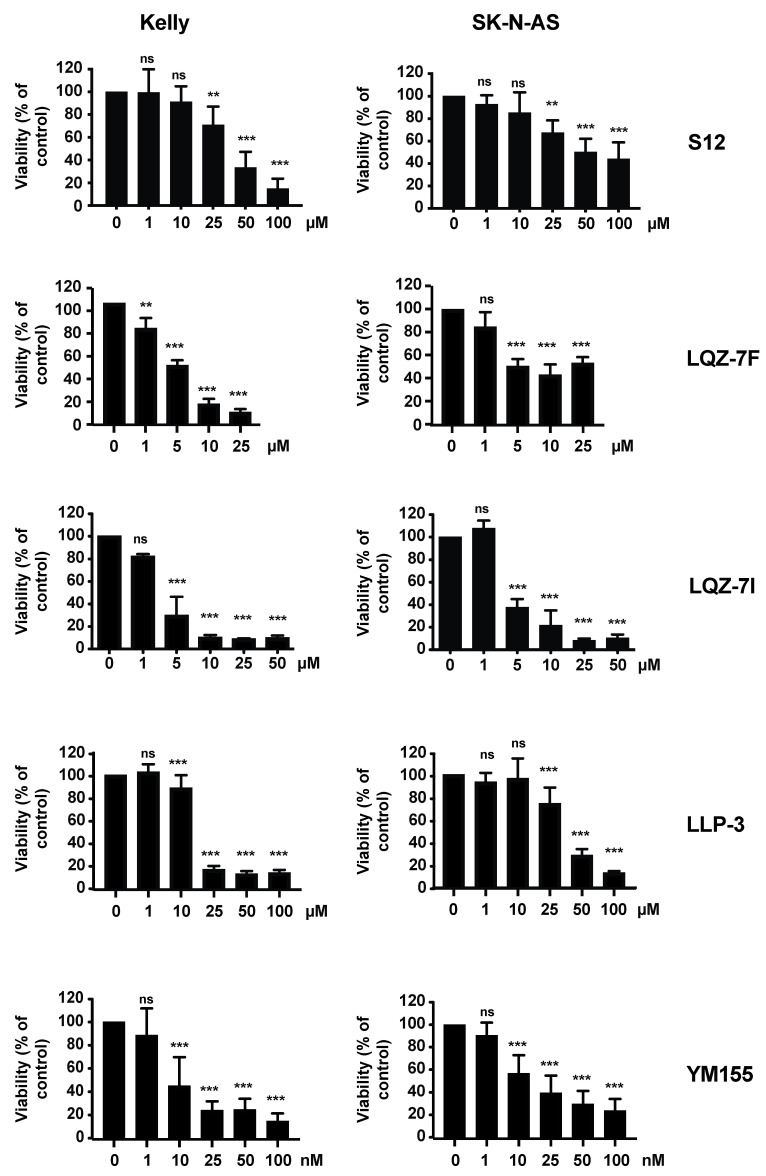
Heterogenous efficacy of survivin inhibitors on NB cell viability. NB cells were seeded in 96-well plates and treated with inhibitors at indicated concentrations for 72 h. MTT viability assay was performed. Absorption values were normalized to DMSO-treated controls. Shown are the means and SD of three independent experiments performed at least in triplicates. Statistical analysis was performed using the ANOVA test. ns, not significant; ** *p* < 0.01; *** *p* < 0.001.

As the inhibition of survivin transcription did not result in control of NB, we performed orienting experiments with inhibitors of survivin protein interactions. To this end, we focused on the survivin homodimerization inhibitors S12, LQZ-7F and LQZ-7I, and compared them with the survivin heterodimerization inhibitor LLP-3 and the survivin transcription inhibitor YM155. The paradigmatic NB cell lines Kelly (*MYCN*-amplified and p53 wild-type) and SK-N-AS (*MYCN* non-amplified and p53 mutated) were assessed in vitro. The homo- and heterodimerization inhibitors decreased viability when used in the micromolar range, more so in Kelly than in SK-N-AS cells (Figure 3). S12, LQZ-7I and LLP-3 had comparable efficacy, while LQZ-7F was less efficacious. YM155 was effective in nanomolar concentrations (Figure 3).

### 3.4. The Survivin Dimerization Inhibitor S12 Decreases Growth of NB Cells

**Figure 4:**
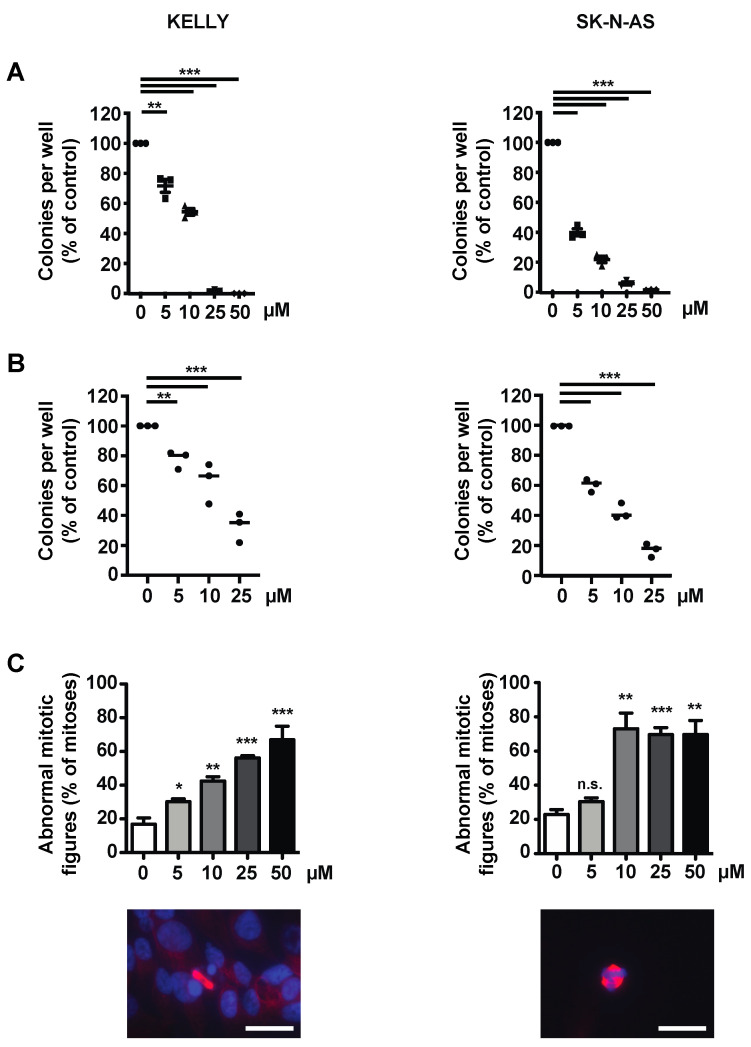
S12 decreases the viability, clonogenicity, anchorage-independent growth and correct mitosis of NB cell lines. Kelly and SK-N-AS cells were treated with increasing concentrations of S12. Controls were treated with 0.1% DMSO in PBS. (**A**) Dose-dependent decrease of clonogenicity. Kelly and SK-N-AS cells were seeded in clonal density into 6-well plates. Colonies were stained with crystal violet and counted. Shown are means of two independent experiments performed in triplicates. (**B**) Dose-dependent reduction of anchorage-independent growth. Kelly and SK-N-AS were seeded at clonal density into soft agar. Colonies were stained with MTT solution and counted. Shown are the means of two independent experiments performed in dodecaplicates. (**C**) Dose-dependent abnormalities of mitotic figures. Kelly and SK-N-AS cells were seeded in 24-well plates. Formalin-fixed samples were stained for α-Tubulin (red) and DNA (blue). The upper panel shows the quantification of abnormal mitotic figures counted, presented as a percentage of total mitotic figures. The lower panel depicts representative cells with abnormal mitotic figures. Bars equal 25 μm. Experiments were performed in duplicates and repeated three times. Shown are the means of three independent experiments. Statistical analysis of all experiments was performed using the ANOVA test. n.s., not significant; * *p* < 0.05; ** *p* < 0.01; *** *p* < 0.001.

Given that YM155 had failed in clinical trials, despite promising preclinical results, and since S12 and LQZ-7I showed efficacy in our orienting experiments, we decided to investigate these compounds in more detail. S12 attenuated clonogenicity and anchorage-independent growth of Kelly and SK-N-AS cells (Figure 4A,B) and of other *MYCN*-amplified and non-amplified NB cell lines (Supplementary Figure S2). Kelly and SK-N-AS cells treated with increasing doses of S12 showed increasing numbers of abnormal mitotic figures, as indicated by multiple or disarranged mitotic spindles (Figure 4C).

It was conceivable that S12, in addition to inhibiting homodimerization, may inhibit the heterodimerization of survivin. The GTPase Ran binds near the homodimerization domain of survivin [13], close to the position where S12 interacts with survivin [29]. Thus, we investigated whether S12 inhibits heterodimerization with Ran. This was not the case, as shown by proximity ligation assay (Supplementary Figure S3).

Taken together, the presumptive homodimerization inhibitor S12 inhibits the growth of both *MYCN*-amplified and non-amplified NB cells in vitro, most likely by inducing errors in spindle formation and thus mitosis.

### 3.5. S12 Decreases Intratumoral Hemorrhage of Subcutaneous Kelly NB Xenografts While Not Inhibiting Their Growth

**Figure 5:**
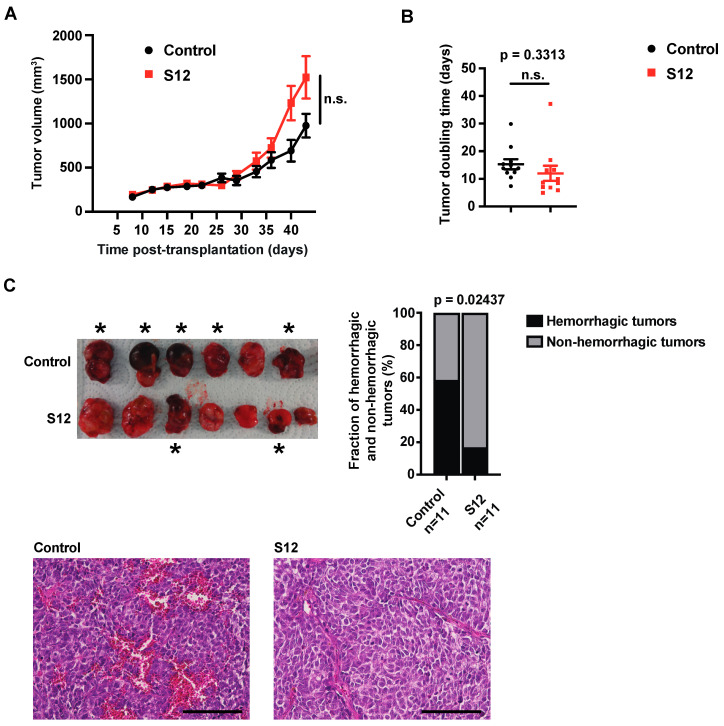
S12 decreases intratumoral hemorrhage in Kelly NB xenografts while not attenuating their growth. 2 × 10^6^ Kelly cells were injected subcutaneously into the left flank of athymic mice. Seven days later, mice received intraperitoneal injections three times per week with 30 mg/kg of S12 while the control group received vehicle (40% PEG in PBS) only. Eleven animals per group were investigated. (**A**) S12 does not decrease growth of Kelly NB xenografts. The tumor volumes of treated and control groups over the duration of treatment are shown. The ANOVA test was used for statistical analysis. ns., not significant. (**B**) S12 does not increase doubling time. Tumor-doubling times were calculated. Statistical analysis was performed using the unpaired *t*-test. ns., not significant. (**C**) S12 decreases intratumoral hemorrhage. Tumors were inspected visually after excision and scored as hemorrhagic or non-hemorrhagic. The upper left panel shows representative tumors. Asterisks mark hemorrhagic tumors. For statistical analysis the Chi-square test was used (upper right panel). The lower panel depicts representative H&E-stained histological slides (bars equal 100 µm).

As S12 was efficacious against NB in vitro, we investigated its efficacy in vivo. To this end, *MYCN*-amplified Kelly cells were injected subcutaneously into athymic nude mice. Once tumors were established, mice were treated systemically with S12 until the end of the experiment. Surprisingly, S12 did not alter tumor growth and doubling time (Figure 5A,B). Interestingly, the treated tumors appeared less hemorrhagic upon inspection and histological analysis (Figure 5C). Tumor proliferation, apoptosis and vascularization, as determined by Ki67, activated caspase-3 and CD31 staining, respectively, were not altered upon S12 treatment (Supplementary Figure S4).

As little is known about side effects of S12, toxicity was assessed in non-tumor bearing mice receiving S12 for 4 weeks. No weight loss was observed (Supplementary Figure S5A). Mild hematological toxicity with decreased levels of red blood cells, neutrophils and platelets was evident (Supplementary Figure S5B). Necropsy and histological analysis of major organs revealed no abnormalities.

Taken together, while decreasing intratumoral hemorrhage and causing only mild hematological toxicity, S12 did not attenuate NB cell growth in vivo.

### 3.6. The Survivin Dimerization Inhibitor LQZ-7I Abrogates NB Cell Growth

Since S12 had limited efficacy against NB tumors, and because LQZ-7I had shown promising results in our orienting experiments (Figure 3), we assessed LQZ-7I in more depth. Of note, the viability of Kelly and SK-N-AS cells treated with LQZ-7I started to decrease at lower drug concentrations and was more pronounced at a given concentration when compared to S12 (Figure 3).

**Figure 6:**
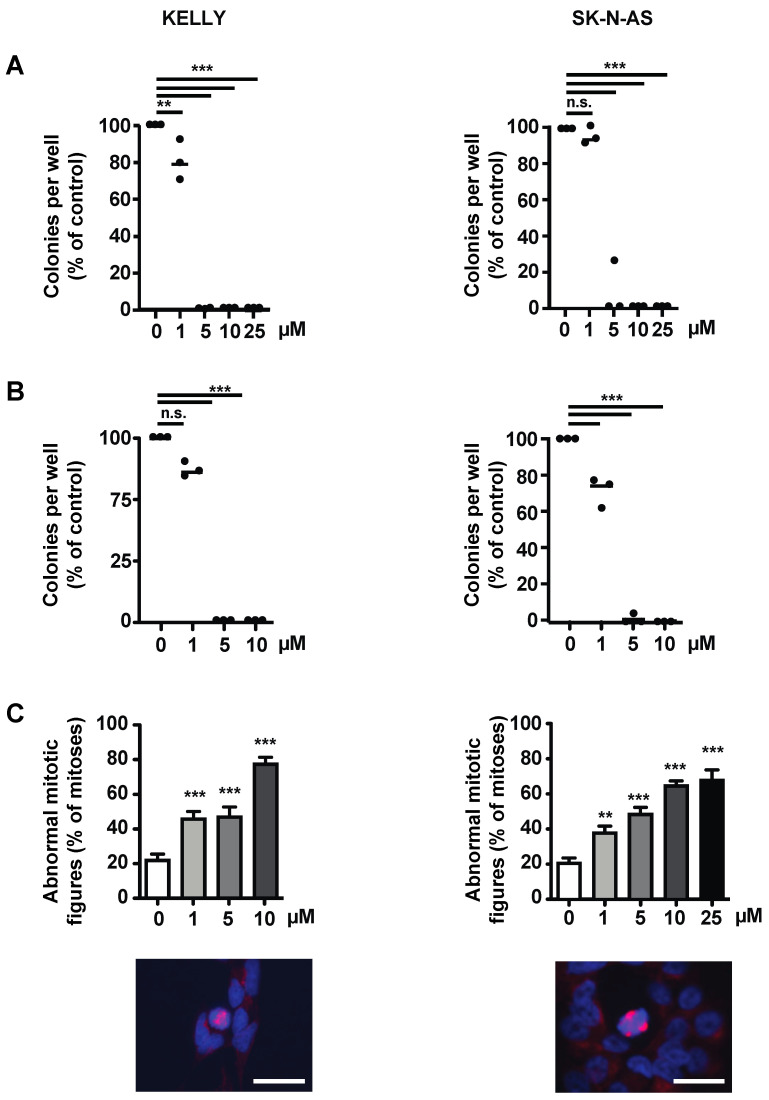
LQZ-7I abrogates viability, clonogenicity and anchorage-independent growth, and inhibits correct mitosis of NB cell lines. Kelly and SK-N-AS cells were treated with increasing concentrations of LQZ-7I. Controls were treated with 0.1% DMSO in PBS. (**A**) Dose-dependent abrogation of clonogenicity. Kelly and SK-N-AS cells were seeded in clonal density into plastic wells. Colonies were stained with crystal violet and counted. Shown are the means of two independent experiments performed in triplicates. (**B**) Dose-dependent abrogation of anchorage-independent growth. Kelly and SK-N-AS were seeded at clonal density in soft agar and treated with the indicated concentrations of LQZ-7I. Colonies were stained with MTT solution and counted. Shown are the means of two independent experiments performed in dodecaplicates. (**C**) Dose-dependent abnormalities of mitotic figures. Kelly and SK-N-AS cells were seeded in 24-well plates. Formalin-fixed samples were stained for α-Tubulin (red) and DNA (blue). The upper panel shows the quantification of abnormal mitotic figures, presented as a percentage of total mitotic figures. The lower panel depicts representative cells with abnormal mitotic figures. Bars equal 25 μm. Experiments were performed in duplicates and repeated three times. Shown are the means of three independent experiments. Statistical analysis of all experiments was performed using the ANOVA test. n.s., not significant; ** *p* < 0.01; *** *p* < 0.001.

Low micromolar concentrations of LQZ-7I sufficed to abrogate clonogenicity of Kelly and SK-N-AS cells (Figure 6A). This was also evident in a large number of additional *MYCN*-amplified and non-amplified NB cell lines (Supplementary Figure S6), with the latter being somewhat more sensitive.

Anchorage-independent growth of Kelly and SK-N-AS cells was also abrogated already at a dose of 5 µM (Figure 6B). LQZ-7I induced a large number of abnormal mitotic figures depending on dose (Figure 6C). The abnormal mitotic figures were similar to the abnormal patterns induced by S12 treatment (Supplementary Figure S7).

In summary, LQZ-7I efficiently abrogates the clonogenicity, anchorage-independent growth and viability of NB cells by inducing markedly abnormal mitoses.

### 3.7. LQZ-7I Attenuates the Proliferation of SK-N-AS Cells In Vivo in the Absence of Toxicity

**Figure 7:**
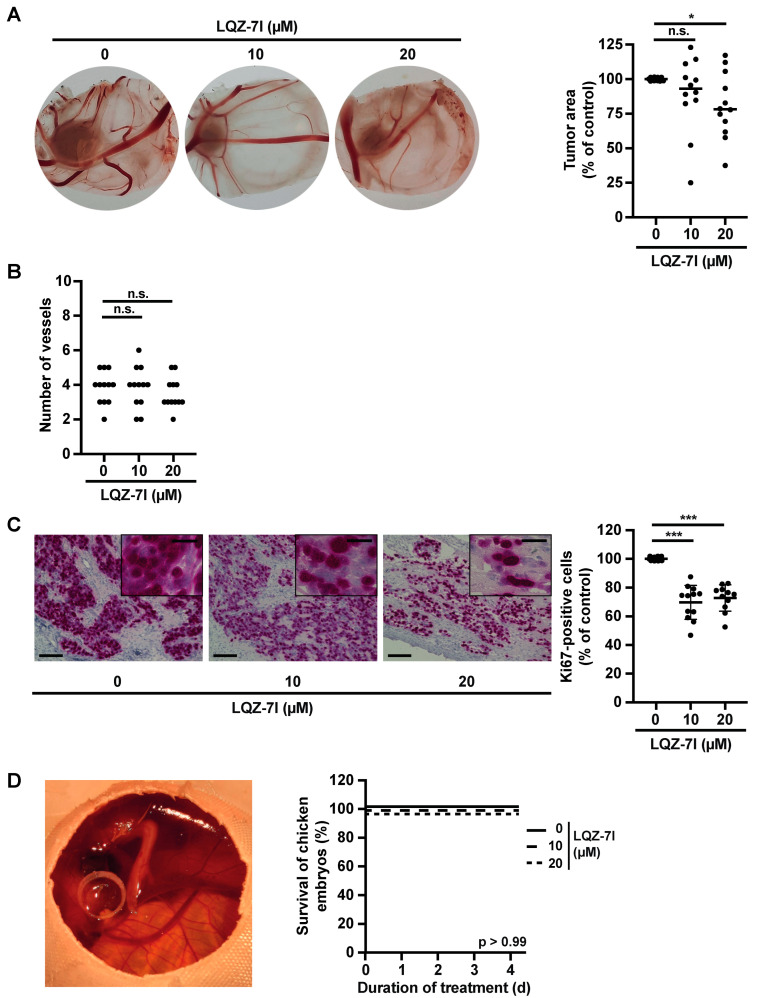
LQZ-7I decreases growth of SK-N-AS cells in ovo without embryonic toxicity. SK-N-AS cells were seeded onto chorioallantoic membranes (CAMs) of fertilized chicken eggs. Tumors were treated from day 1 post-seeding twice per day for 3 days with the indicated concentrations of LQZ-7I. On day 4, CAMs with tumors were excised. Two independent experiments were performed in hexaduplicates. (**A**) LQZ-7I decreases tumor size. Tumor area was measured and expressed as a percentage of the control. Means were analyzed using a one-tailed *t*-test. n.s., not significant; 20 µM * *p* < 0.05. (**B**) LQZ-7I does not decrease angiogenesis. Macroscopically visible blood vessels on the tumors were counted. (**C**) LQZ-7I decreases proliferation. Tumors were stained for Ki67 by immunohistochemistry. Ki67-positive cells were counted at 20× magnification in two visual fields per tumor. Representative images are shown in the left panel, with bars representing 100 μm and with bars in inserts representing 25 µm. The graph shows the percentage of Ki67-positive cells compared to the untreated control. Means were analyzed using a one-tailed *t*-test. *** *p* < 0.001. (**D**) LQZ-7I does not affect survival of developing embryos. Survival was determined by inspecting movements of the embryos visible below the CAM. Shown are Kaplan–Meier survival curves for the duration of the experiments. Statistical analysis was performed using the log rank test. n.s., not significant.

Given the efficacy of LQZ-7I against NB in vitro, we investigated its efficacy in vivo using the chorioallantoic membrane (CAM) assay. We chose this model due to its immune tolerance, visible angiogenesis, and ability to assess the toxicity of LQZ-7I in the highly sensitive developing chick embryo. Following three days of treatment, LQZ-7I significantly reduced tumor size (Figure 7A). The morphology and number of tumor blood vessels remained unaltered (Figure 7A,B). Notably, LQZ-7I decreased the density of NB cells and markedly diminished the proportion of proliferating, or Ki67-positive, tumor cells (Figure 7C). All embryos survived until the experiment’s conclusion (Figure 7D). Collectively, these findings demonstrate that LQZ-7I effectively reduces tumor size and cell proliferation of SK-N-AS cells in vivo without apparent toxicity to the developing chick embryo.

## 4. Discussion

This work addressed the hitherto under-researched efficacy of targeting survivin in NB. In a novel transgenic NB mouse model with *Birc5* heterozygosity we show that a marked, albeit incomplete, prevention of survivin transcription did not suffice to decrease NB growth. Conversely, inhibiting survivin homodimerization using the homodimerization inhibitors S12 and LQZ-7I effectively controlled NB cells in vitro, and LQZ-7I also demonstrated efficacy in vivo.

YM155 has been described to be effective in NB in vitro [41]. We have confirmed this in SK-N-AS and Kelly NB cells. However, in vivo studies of YM155 in NB have led to contradictory results [28,42]. Along this line, YM155 failed in multiple early phase clinical trials in various cancers due to insufficient antitumor efficacy [22,23,24,25,28,43,44,45,46,47,48]. This may be explained by insufficient stability and incomplete specificity of the compound [17]. Inhibiting the transcription of oncogenes for clinical purposes is principally difficult, given the promiscuity of many transcription factors and the challenge of sufficiently decreasing transcription levels. Using a novel *Birc5+/- MYCN tg/*+ NB mouse model, we show that even a specific, i.e., genetic, and marked inhibition of survivin transcription was insufficient to inhibit NB growth, in contrast to the efficacy of YM155 we observed in vitro. The results of this clinically relevant genetic NB mouse model question the feasibility of drugs targeting survivin transcription for the treatment of NB. The lack of NB control by survivin inhibition in the transgenic mouse model was not due to the inhibition of survivin in immune cells, as transplantation of NB from *Birc5*+/- *MYCN tg*/+ mice into wild-type mice did not decrease tumor growth. Since the proliferation of T cells requires survivin [38,49], this suggests that an incomplete systemic inhibition of survivin may not decrease the anti-tumor function of T cells.

Inhibiting the homo- or heterodimerization of survivin, rather than targeting its transcription, may have the advantage of increased specificity because many transcription-related targets, such as transcription factors, transcription factor drivers, promoter elements or epigenetic modifiers, influence the transcription of multiple genes.

In vitro experiments with LLP-3, which inhibits the heterodimerization of survivin with the GTPase Ran [35], were performed to serve as a comparison for the effectiveness of the homodimerization inhibitors S12 and LQZ-7I, which were the focus of this study. The preliminary results show promise for the use of LLP-3 against NB, warranting further investigations of this compound in NB.

This work provides evidence that small molecules disrupting survivin-survivin interaction can inhibit NB cells in vitro. Thus, S12, which was designed to inhibit survivin homodimerization [29] but may also inhibit heterodimerization [17], controlled NB cells by inducing mitotic dysfunction. In vivo, S12 attenuated the hemorrhagic phenotype of some tumors. There are two implications of a NB with an intratumoral hemorrhage. First, such a tumor may be fragile and therefore harder to remove by the surgeon. Second, the risk of spontaneous rupture, a rare but life-threatening complication of NB [50], may be increased. It is tempting to speculate that S12 may have decreased survivin-mediated vascular integrity in these tumors [51]. While the general toxicity of S12, as determined by weight loss and solid organ toxicity, was absent, and hematopoietic toxicity was moderate, S12 did not control NB growth in vivo. There are several possible explanations for this treatment failure. First, the drug concentrations in the high micromolar range that were required to control the NB cells in vitro may not have been achieved in vivo. This may have been caused by the dose having been too low or the application interval having been too long. Second, S12 may not have sufficiently entered the tumor due to insufficient or heterogenous tumor vascularization, or impaired evasion from the tumor vasculature into the tumor. Third, the use of a non-orthotopic and non-metastatic NB tumor model in immunodeficient NB mice lacking anti-tumor immune cells may have masked the efficiency of S12.

LQZ-7I is a recently developed and specific survivin homodimer inhibitor [32]. We show that LQZ-7I is efficacious against NB cells, more so than the structurally similar compound LQZ-7F. Compared to S12, LQZ-7I more efficiently inhibited viability, clonogenicity and anchorage-independent growth. The observed strong impairment of mitotic function by LQZ-7I is in line with its described molecular action of inhibiting survivin homodimerization and consequent survivin degradation [31]. In vivo administration of LQZ-7I for a short period proved sufficient to reduce tumor size and proliferation of SK-N-AS neuroblastoma (NB) cells in the CAM assay, without alterations in large tumor vessels and without discernible toxicity to the developing chick embryo. The duration of therapy and observation was restricted due to animal welfare regulations. We hypothesize that prolonged administration and observation would have further amplified the inhibitory effects of LQZ-7I on tumor size and proliferation, and would have enabled the elucidation of its effects on invasion and metastasis. There are limitations of the CAM model. It largely precludes intravenous administration of drugs and thus analysis of their effect on cancers. In addition, the microenvironment of the CAM differs from that of humans. Despite these limitations, the in vivo results support the conclusion that LQZ-7I is effective against NB.

## 5. Conclusions

Inhibitors of survivin homo- and heterodimerization, including LQZ-7I, demonstrate efficacy against NB cells. Targeting survivin protein interactions appears to be more effective in controlling NB cell growth compared to incomplete inhibition of survivin transcription. These findings suggest that survivin dimerization inhibitors represent a promising new therapeutic approach for NB, warranting further investigation.

## Data Availability

All data needed to evaluate the conclusions of this study are present in the paper.

## Supplementary Materials

The following supporting information can be downloaded at: https://www.mdpi.com/article/10.3390/cancers15245775/s1, Supplementary Materials and Methods; Figure S1: Hemizygosity of *Birc5* does not alter proliferation, apoptosis and angiogenesis of NB; Figure S2: S12 decreases clonogenicity of *MYCN*-amplified and non-amplified NB cell lines; Figure S3: S12 does not affect the interaction of Survivin with Ran in NB cells; Figure S4: Systemic S12 does not alter proliferation, apoptosis and angiogenesis of Kelly NB tumors. Figure S5: Limited hematological toxicity by S12. Figure S6: LQZ-7I decreases clonogenicity of *MYCN*-amplified and non-amplified NB cell lines. Figure S7: S12 and LQZ-7I increase frequency of aberrant mitotic figures in NB cells. File S1: Original western blots.
