# Supplementary material for: Inhibition of Survivin Homodimerization Decreases Neuroblastoma Cell Growth
Source: Cancers (Basel). 2023 Dec 9;15(24):5775. doi: 10.3390/cancers15245775 (PMC10741502; doi:10.3390/cancers15245775)
Supplement: Supplementary file 1 [file cancers-15-05775-s001.zip › File S1.pdf]

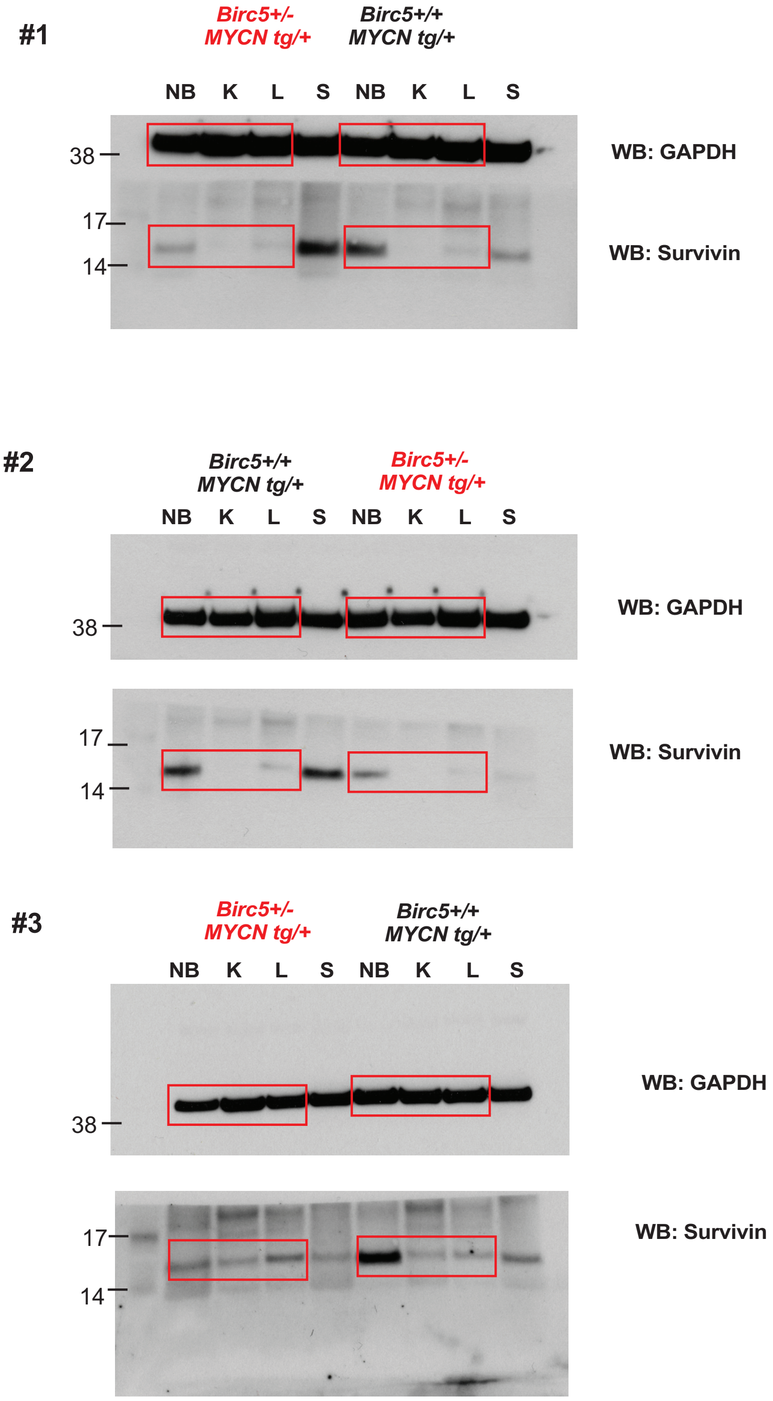


***Original Western blots of Fig. 1E.***

***Hemizygous deletion of Birc5 markedly decreases survivin protein levels.*** *Proteins were isolated form tumors (NB), kidneys (K), livers (L) and spleens (S) of Birc5+/+MYCN tg/+ and Birc5+/-MYCN tg/+ mice. Survivin protein levels were determined by Western blot. The red boxes mark the results presented in the manuscript. Shown are the results of 3 pairs of littermates (#1, #2 and #3).*
