# Supplementary material for: Inhibition of Survivin Homodimerization Decreases Neuroblastoma Cell Growth
Source: Cancers (Basel). 2023 Dec 9;15(24):5775. doi: 10.3390/cancers15245775 (PMC10741502; doi:10.3390/cancers15245775)
Supplement: Supplementary file 1 [file cancers-15-05775-s001.zip › Suppl Figure S1.pdf]

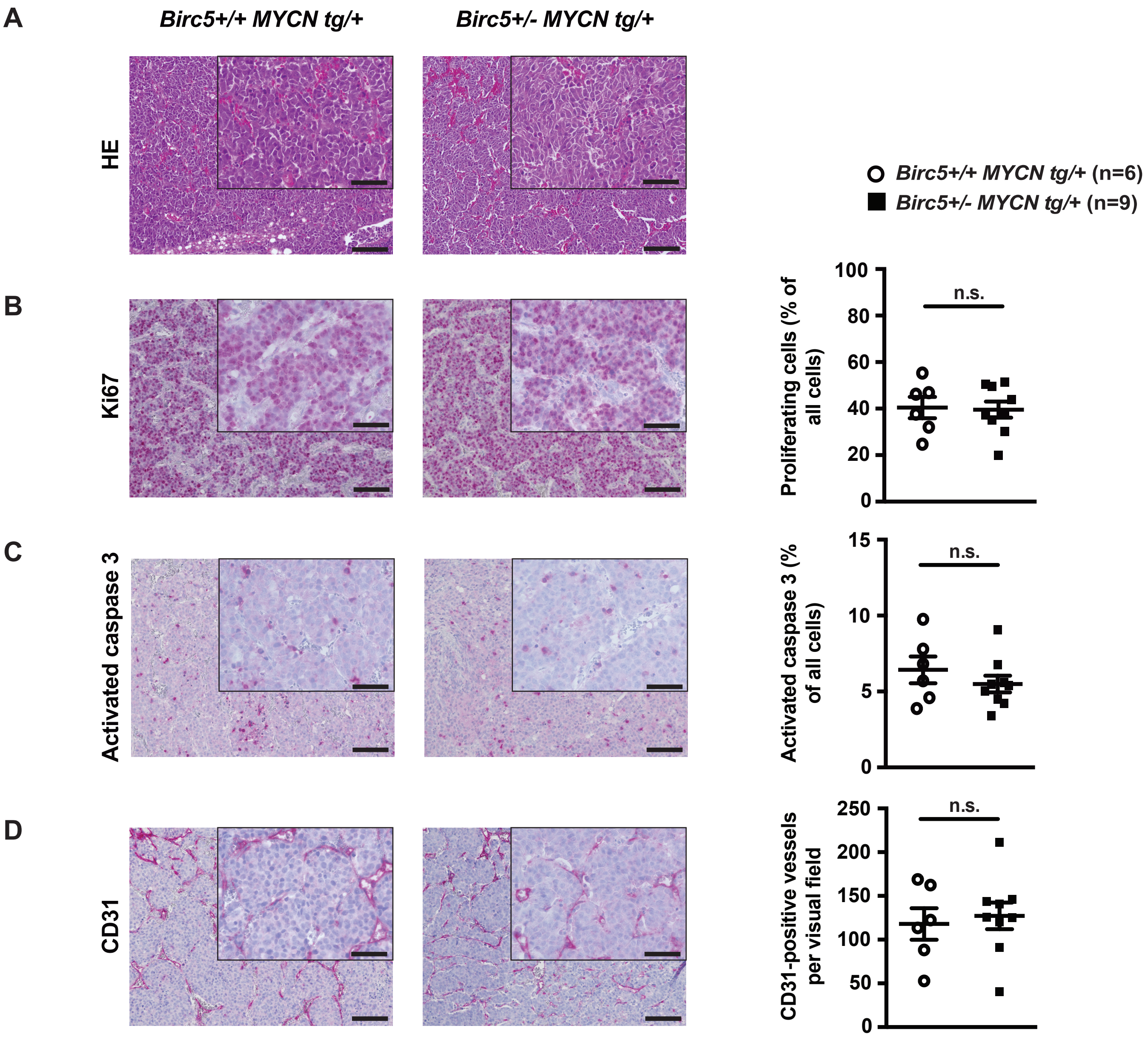

Suppl. Figure S1: Hemizygosity of Birc5 does not alter proliferation, apoptosis and angiogenesis of NB. *Tumors were formalin-fixed, paraffin-embedded, stained and analyzed. Scale bars equal 100 µm and 50µm (insert). Statistical analysis was performed using the unpaired two-tailed t-test. n.s, not significant.* ***A) Hemizygosity of Birc5 does not alter tumor tissue.*** *Shown is one representative picture of H&E stained tumors per genotype.* ***B) Proliferation of tumor cells is not decreased by hemizygosity of Birc5.*** *Representative Ki67 stains are shown in the left panel, respectively. Ki67-positive cells were counted as % of nucleated cells in 3-4 representative visual fields (x40) per tumor and are depicted as means and standard deviations (right panel).* ***C) Hemizygosity of Birc5 does not increase apoptosis rate in the tumors.*** *Representative active caspase 3 stains are shown in the left panel, respectively. Active caspase 3-positive cells were counted as % of nucleated cells in 3-4 representative visual fields (x40) per tumor and are depicted as means and standard deviations (right panel).* ***D) Hemizygosity of Birc5 does not attenuate angiogenesis in the tumors.*** *Representative CD31 stains are shown in the left panel, respectively. Scale bars equal 100 µm. CD31-positive vessels were counted in 4-5 representative visual fields (x20) per tumor and are depicted as means and standard deviations (right panel).*
