# Supplementary material for: Inhibition of Survivin Homodimerization Decreases Neuroblastoma Cell Growth
Source: Cancers (Basel). 2023 Dec 9;15(24):5775. doi: 10.3390/cancers15245775 (PMC10741502; doi:10.3390/cancers15245775)
Supplement: Supplementary file 1 [file cancers-15-05775-s001.zip › Suppl figure S2.pdf]

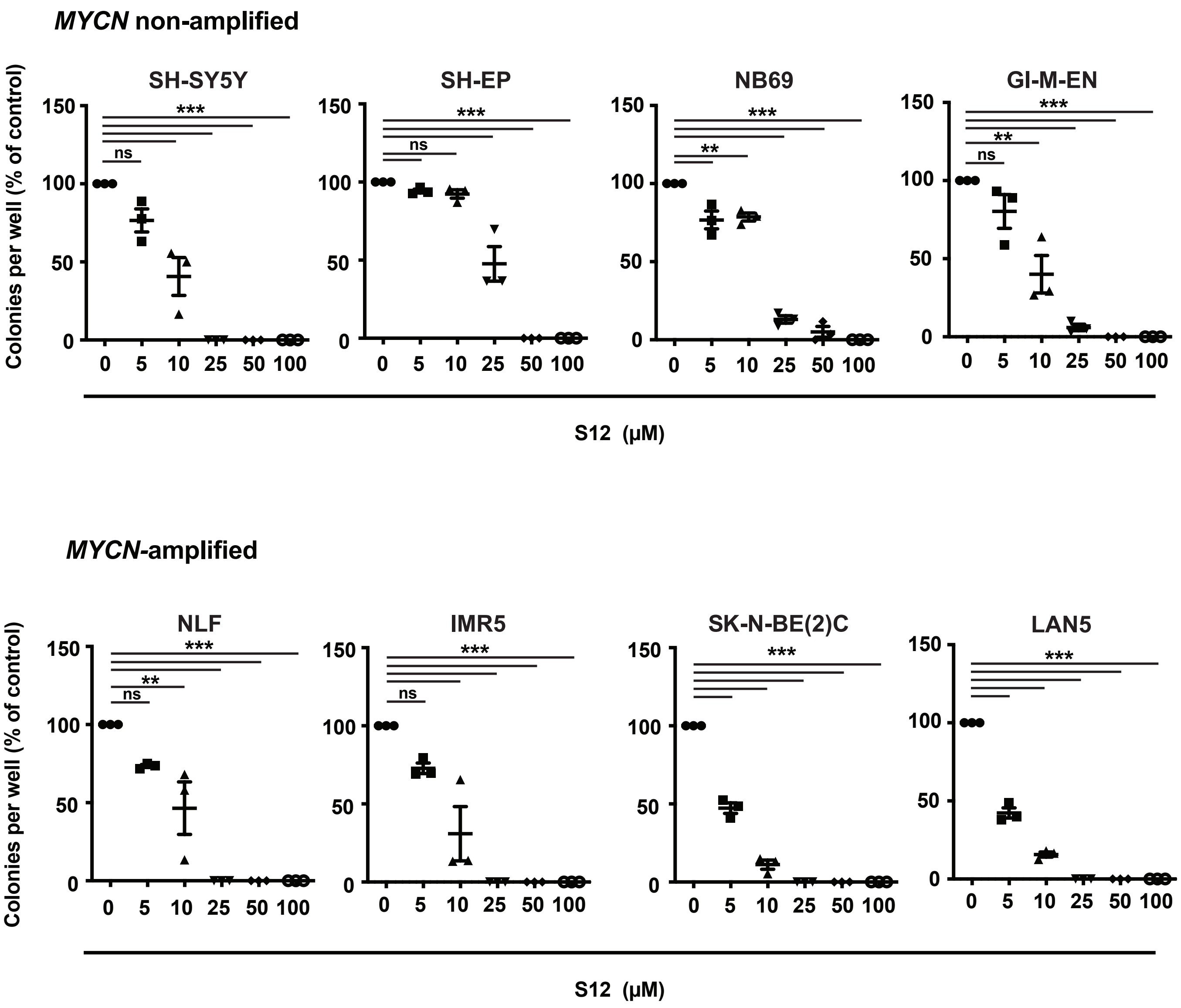

Suppl. Fig. S2: ***S12 decreases clonogenicity of MYCN-amplified and non-amplified NB cell lines.*** *MYCN non-amplified (upper panel) and MYCN-amplified (lower panel) NB cells were seeded in clonal density into plastic cells and treated with indicated concentrations of S12. Colonies were stained with crystal violet and counted. Shown are the means of three independent experiments performed in triplicates. Statistical anlysis was performed using the one-way ANOVA test. ns, not significant; * p<0.05; **p<0.01; ***p<0.001.*
