# Supplementary material for: Inhibition of Survivin Homodimerization Decreases Neuroblastoma Cell Growth
Source: Cancers (Basel). 2023 Dec 9;15(24):5775. doi: 10.3390/cancers15245775 (PMC10741502; doi:10.3390/cancers15245775)
Supplement: Supplementary file 1 [file cancers-15-05775-s001.zip › Suppl figure S3.pdf]

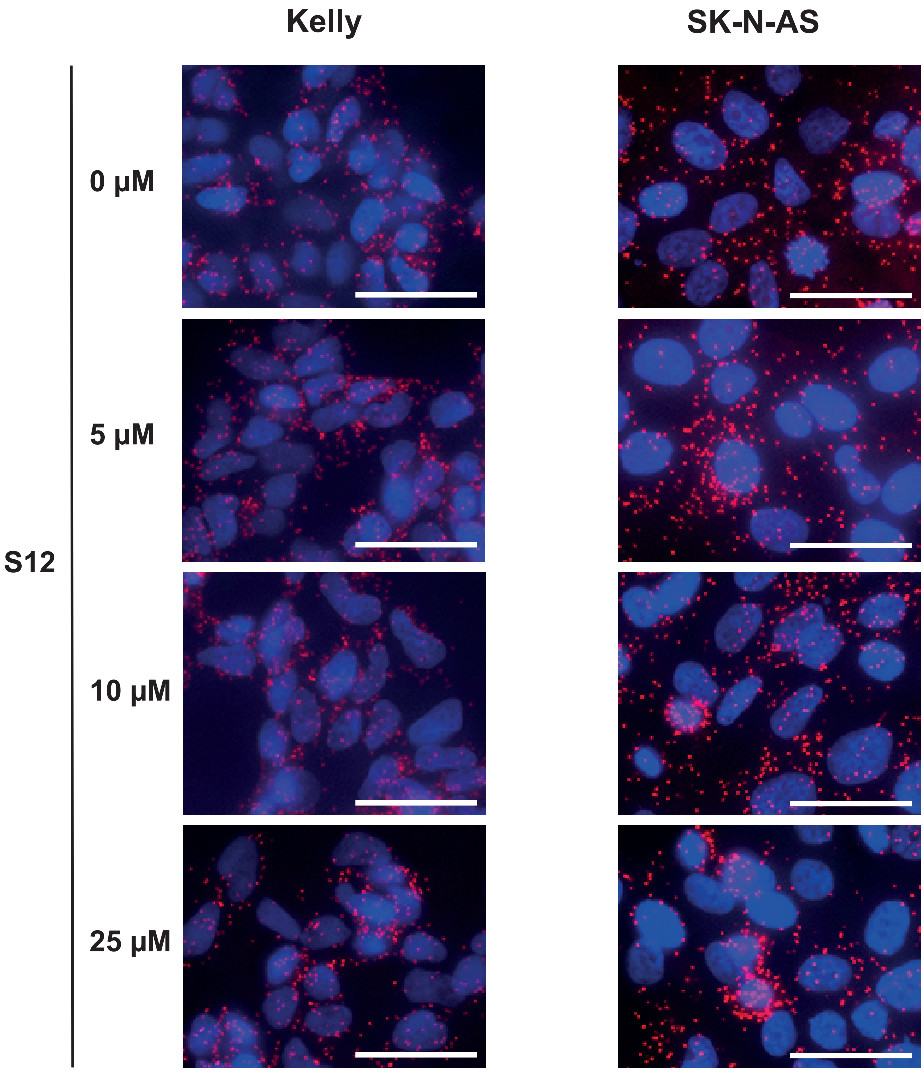

Suppl. Fig. S3: S12 does not affect the interaction of Survivin with Ran in NB cells. *NB cells were seeded onto chamber slides and treated with S12 or with DMSO as vehicle control, respectively. After 24h cells were fixed and proximity ligation assay was performed. Shown are representative pictures from a total of three independent experiments performed in duplicates. Bars equal 30µm.*
