# Supplementary material for: Inhibition of Survivin Homodimerization Decreases Neuroblastoma Cell Growth
Source: Cancers (Basel). 2023 Dec 9;15(24):5775. doi: 10.3390/cancers15245775 (PMC10741502; doi:10.3390/cancers15245775)
Supplement: Supplementary file 1 [file cancers-15-05775-s001.zip › Suppl figure S4.pdf]

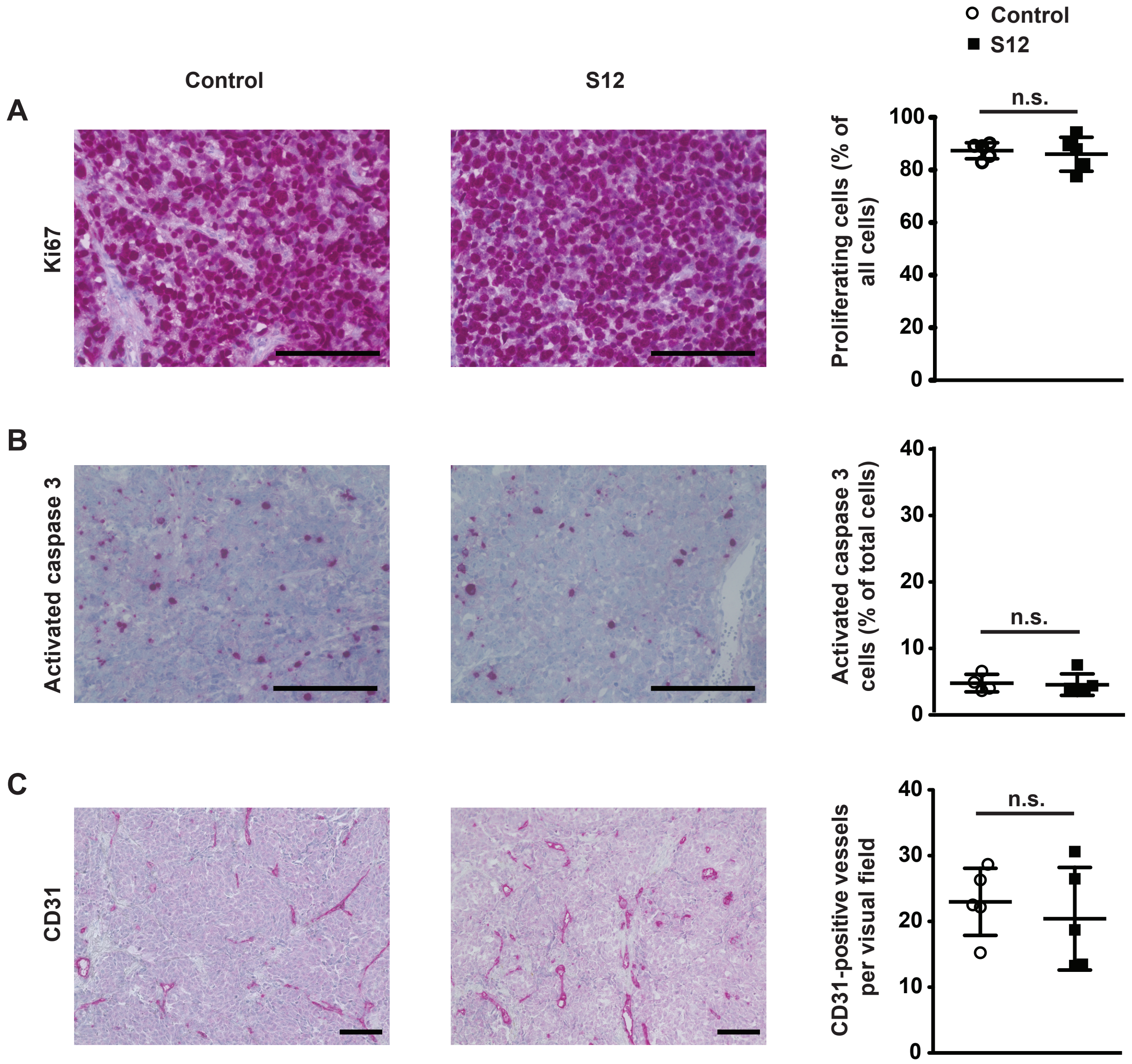

Suppl. Figure S4: Systemic S12 does not alter proliferation, apoptosis and angiogenesis of Kelly NB tumors. *2x10^6 Kelly cells were injected s.c. into the left flank of athymic mice. After 7 days mice received i.p. injections three times per week with 30 mg/kg of S12 while control group received PBS with 40% PEG. Tumors of 4-5 mice per group were formalin-fixed, paraffin-embedded, stained and analyzed.* ***A) Proliferation of Kelly cells is not affected by S12 treatment.*** *Representative Ki67 stains are shown in the left panel, respectively. Ki67-positive cells were counted as % of nucleated cells in 5-9 representative visual fields (x40) and are depicted as means and standard deviations (right panel).* ***B) S12 treatment does not induce apoptosis in Kelly tumors.*** *Representative active caspase 3 stains are shown in the left panel, respectively. Active caspase 3-positive cells were counted as % of nucleated cells in 5-7 representative visual fields (x40) and are depicted as means and standard deviations (right panel).* ***C) Systemic injection of S12 does not alter tumor vessel formation.*** *Representative CD31 stains are shown in the left panel, respectively. CD31-positive vessels were counted in 4-8 representative visual fields (x20) and are depicted as means and standard deviations (right panel).*

Statistical analysis was performed using the unpaired two-tailed t-test. n.s, not significant. Scale bars equal 100 µm.
