# Supplementary material for: Inhibition of Survivin Homodimerization Decreases Neuroblastoma Cell Growth
Source: Cancers (Basel). 2023 Dec 9;15(24):5775. doi: 10.3390/cancers15245775 (PMC10741502; doi:10.3390/cancers15245775)
Supplement: Supplementary file 1 [file cancers-15-05775-s001.zip › Suppl figure S5.pdf]

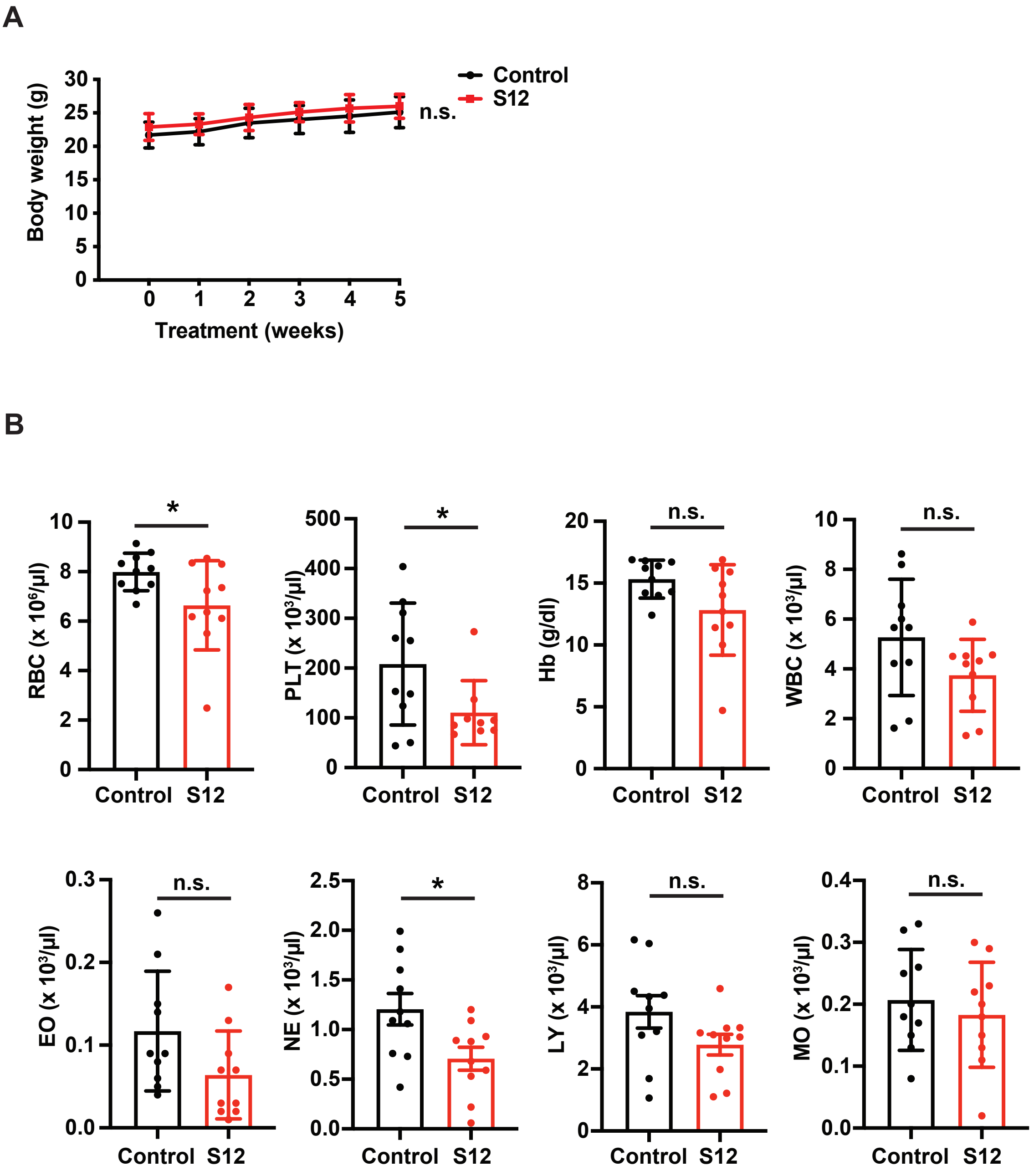

Suppl. Figure S5: Limited hematological toxicity by S12. *Six-week-old athymic mice (n=5 per group) were treated intraperitoneally with 30 mg/kg of S12 three times per week for 4 weeks. Control animals received only vehicle (PBS with 40 % PEG).* ***A) Mouse body weight is not affected by systemic S12 injection.*** *Body weight was measured once per week for the duration of the experiment. 2-way ANOVA was used for statistical analysis.* ***B) Mild hematological toxicity by S12 in athymic nude mice.*** *Blood samples were collected from the tail vein and hematological analysis was performed using a Hemavet 950 FS hematology analyzer. Unpaired t-test was used for statistical analysis. RBC, red blood cells; PLT, platelets; Hb, hemoglobin; WBC, white blood cells; EO, eosinophils; NE, neutrophils; LY, lymphocytes; MO, monocytes.*
