# Supplementary material for: Inhibition of Survivin Homodimerization Decreases Neuroblastoma Cell Growth
Source: Cancers (Basel). 2023 Dec 9;15(24):5775. doi: 10.3390/cancers15245775 (PMC10741502; doi:10.3390/cancers15245775)
Supplement: Supplementary file 1 [file cancers-15-05775-s001.zip › Suppl figure S6.pdf]

***MYCN* non-amplified**

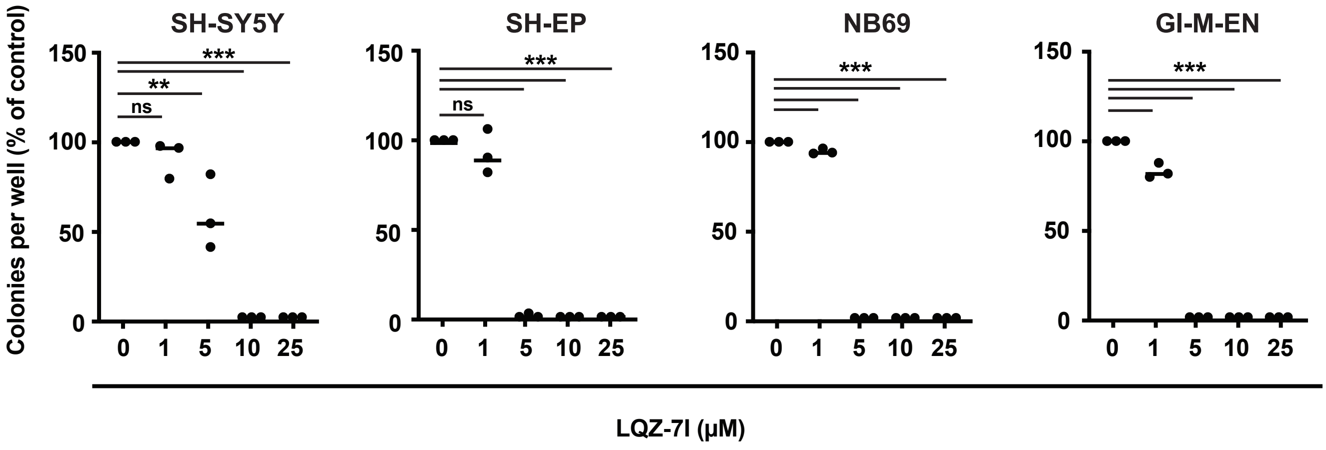

***MYCN*-amplified**

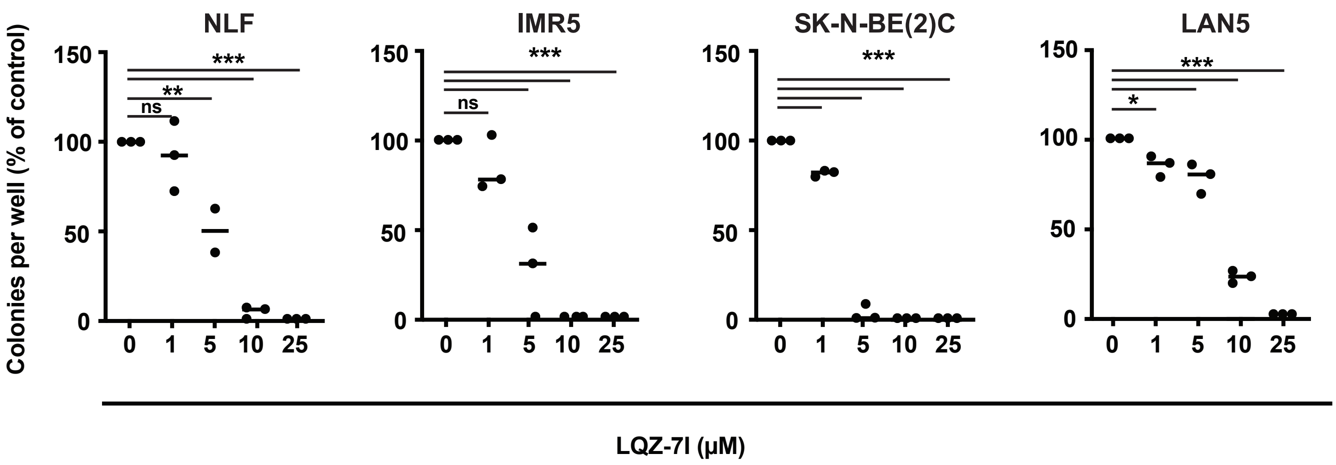

Suppl. Fig. S6: LQZ-7I decreases clonogenicity of MYCN-amplified and non-amplified NB cell lines. *MYCN non-amplified (upper panel) and MYCN-amplified (lower panel) NB cells were seeded in clonal density into plastic cells and treated with indicated concentrations of LQZ-7I. Colonies were stained with crystal violet and counted. Shown are the means of three independent experiments performed in triplicates. Statistical anlysis was performed using the one-way ANOVA test. ns, not significant; * p<0.05; **p<0.01; ***p<0.001.*
