# Supplementary material for: Inhibition of Survivin Homodimerization Decreases Neuroblastoma Cell Growth
Source: Cancers (Basel). 2023 Dec 9;15(24):5775. doi: 10.3390/cancers15245775 (PMC10741502; doi:10.3390/cancers15245775)
Supplement: Supplementary file 1 [file cancers-15-05775-s001.zip › Suppl figure S7.pdf]

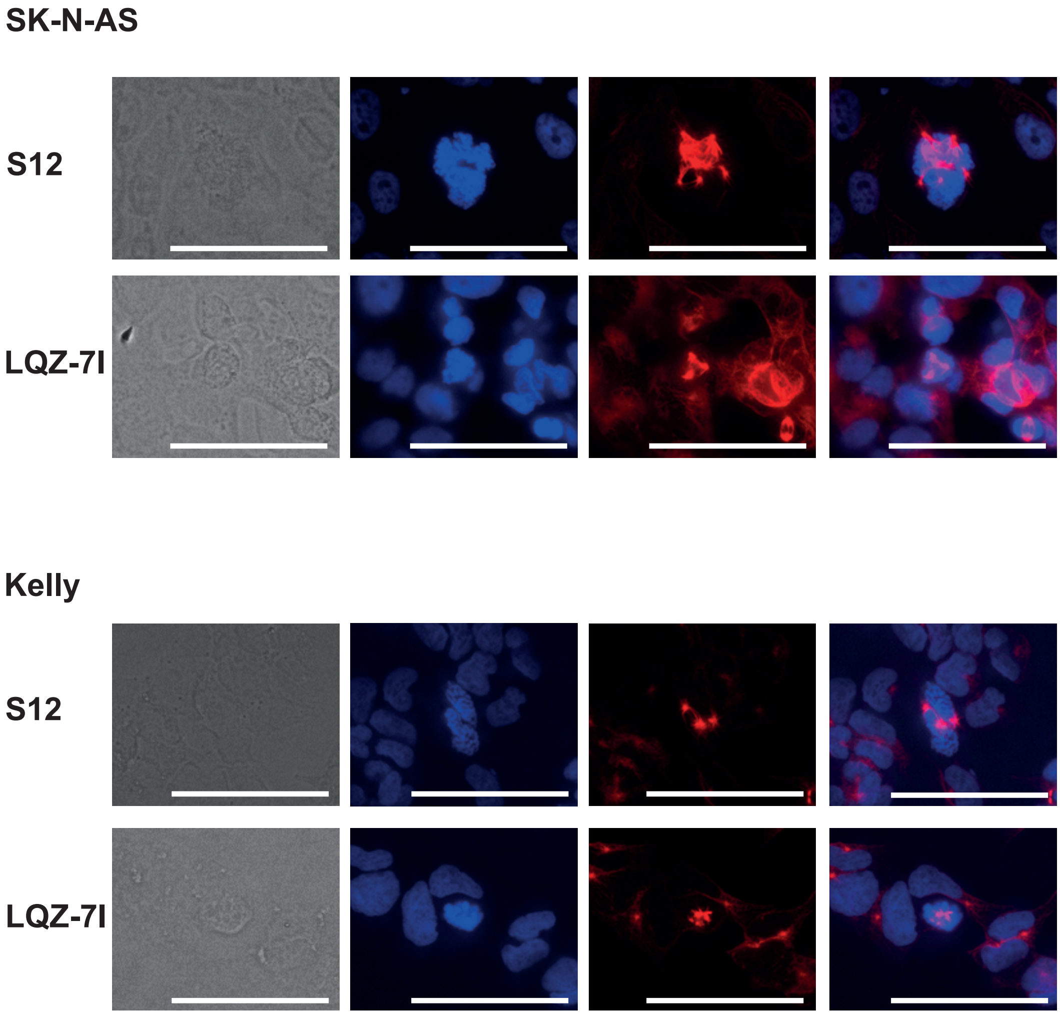

Suppl. Fig. S7: S12 and LQZ-7I increase frequency of aberrant mitotic figures in NB cells. *NB cells were seeded onto cover slips and treated with 10 µM S12 or LQZ-7I, respectively. After 24h cells were fixed and stained for tubulin (red) and DNA (blue). Shown are representative pictures from a total of three independent experiments performed in triplicates. Bars equal 50µm.*
