# Supplementary material for: Inhibition of Survivin Homodimerization Decreases Neuroblastoma Cell Growth
Source: Cancers (Basel). 2023 Dec 9;15(24):5775. doi: 10.3390/cancers15245775 (PMC10741502; doi:10.3390/cancers15245775)
Supplement: Supplementary file 1 [file cancers-15-05775-s001.zip › Supplementary information revision.pdf]

Supplementary information

Supplementary Materials and methods

Immunohistochemistry of CD31

Immunohistochemistry of formalin-fixed, paraffin-embedded sections was performed using standard protocols. Serial sections were deparaffinized and rehydrated. Sections were permeabilized with 0.1% Triton-X100 (Sigma aldrich, T8787) in TBS for 10 min at RT. For antigen retrieval, sections were pressure-cooked in Tris-EDTA buffer pH 9.0, immunoglobulins were blocked with 10 % normal goat serum (Sigma Aldrich G9023) /1 % BSA (bovine serum albumin, Serva, 11930.03)/TBS, followed by incubation with avidin/biotin blocking reagent (DAKO, Hamburg, Germany, X0590) and then probed with rat anti-mouse CD31 (Dianova, Hamburg Germany, dia 310) diluted 1:20 in TBS / 1% BSA at 4°C o/n. Slides were probed with the secondary antibody biotin-SP-conjugated affini-Pure goat anti-rat IgG (H+L) (Dianova, Hamburg, Germany 112-065-003) diluted 1:500 in TBS / 1%BSA for 1h at RT followed by incubation with Alkaline Phosphatase-Streptavidin (Dianova, Hamburg Germany, 016-050-084) diluted 1:750 in TBS. Dako REAL™ Detection System. Alkaline Phosphatase/RED, Rabbit/Mouse (DAKO, Hamburg, Germany, K5005) was used according to the manufacturer´s instruction. Slides were counterstained with Mayer's hematoxylin (Sigma Aldrich, München, Germany, 51275). CD31-positive tumor vessels were counted in 4-8 representative visual fields at x200 magnification. Samples were analyzed using a Keyence BZ-9000 microscope and software.

Immunohistochemistry of Ki67 and active caspase 3

Immunohistochemistry of formalin-fixed, paraffin-embedded sections was performed using standard protocols. Serial sections were deparaffinized and rehydrated. Sections were permeabilized with 0.1% Triton-X100 (Sigma aldrich, T8787) in TBS for 10 min at RT. For antigen retrieval, the sections were pressure-cooked in citrate buffer (10mM, pH 6.0), immunoglobulins were blocked with 10 % normal goat serum (Sigma Aldrich G9023) /1 % BSA (bovine serum albumin, Serva, 11930.03)/TBS, followed by incubation with avidin/biotin blocking reagent (DAKO, Hamburg, Germany, X0590). Slides were probed with antibodies against Ki-67 (MIB-1, Dako, M7240, 1:200) or Cleaved Caspase-3 (Cell Signaling, 9661S, 1:100) diluted in TBS / 1% BSA at 4°C o/n. The slides were probed with the secondary antibody biotin-

SP-conjugated affini-Pure goat anti-rat (IgG (H+L) (Dianova, Hamburg, Germany 112-065-003) diluted 1:500 in TBS / 1%BSA 1h RT followed by incubation of Alkaline Phosphatase-Streptavidin (Dianova, Hamburg Germany, 016-050-084) diluted 1:750 in TBS. Dako REAL™ Detection System, Alkaline Phosphatase/RED, Rabbit/Mouse (DAKO, Hamburg, Germany, K5005) was used according to the manufacturer´s instruction. Slides were counterstained with Mayer's hematoxylin (Sigma Aldrich, München, Germany, 51275). Ki67 and cleaved Caspase 3 postive cells were counted in 4-8 representative visual fields at x400 magnification. Samples were analyzed using a Keyence BZ-9000 microscope and software.

Cell lines and cell culture

The human neuroblastoma cell line Kelly was acquired from DMSZ (Braunschweig, Germany). SK-N-AS was purchased from ATCC (LGC Standard, Wesel, Germany). Kelly cells were cultured in RPMI 1640 (Gibco, Paisley, UK) with 10 % fetal bovine serum (FBS, Gibco), 2 mM glutamine (Gibco) and 100 U/ml penicillin/streptomycin (Gibco). SK-N-AS cells were cultured in DMEM (Gibco) with 10 % FBS, 2 mM glutamine and 100 U/ml penicillin/streptomycin.

Viability assay

NB cell were seeded into 96-well plates and treated with inhibitors. After 72 h cells were treated with 1 mg/ml MTT solution(3-(4,5-dimethylthiazol-2-yl)-2,5diphenyl-tetrazolium bromide, Sigma Aldrich). Formazan crystals formed in viable cells were dissolved by addition of 100 µl isopropanol. Absorbance was measured at 550 nm using an Infinite M plex microplate reader (Tecan, Männedorf, Switzerland). Viability was calculated relative to the DMSO-treated controls.

Soft agar assay

0.6% soft agar was prepared using low melting point agarose (Thermo Fisher Scientific) in growth medium containing FBS and additives. A single cell suspension of 1000 cells/ml in 0.4% top agar was added onto the bottom agar in 24-well plates. Growth medium with S12 was replaced twice a week until colony formation was observed. Colonies were stained with 1 mg/ml MTT (Sigma Aldrich).

Clonogenic growth assay

A single cell suspension of NB cells in clonal density, i.e. 750 cells/well, was seeded in 6-well plates. Growth medium with S12 was added 24h after seeding and replaced once after 72 h with fresh S12. Colonies were stained with crystal violet (Sigma-Aldrich).

Proximity ligation assay

NB cells ($8*10^4$ cells per well) were seeded into 8-well chamber slides (Corning, Kaiserslautern, Germany) previously coated with Collagen I (Corning). Cells were treated with 0, 5, 10 and 25 µM S12. After 24 h, cells were washed twice with PEM buffer (80mM PIPES, 5mM EGTA, 1mM $MgCl_2$, pH 7.4), fixed with 4% PFA in PEM for 15 min, incubated 15 min in PEMT (PEM, 0.2% Tween-20). Samples were washed twice with PEM. Proximity ligation assay was performed using the NaveniFlex MR kit (#NAV.-NF.MR.100, Navinci, Uppsala, Sweden). Primary antibodies were rabbit anti-survivin (1:200, Proteintech #10508-1-AP) and mouse anti-RAN (1:500, Thermo Fisher scientific #MA1-20581). Samples were analyzed using a Keyence BZ-9000 microscope and software.
